# The International Medical Annual

**Published:** 1902-07

**Authors:** 


THE INTERNATIONAL MEDICAL ANNUAL.—A year-book of treatment and practitioners' index. Twentieth year: 1902. Thirty-six contributors, among whom are some of the foremost physicians and surgeons of the United States and Great Britain. Illustrated by twenty-five handsome lithographic plates and sixty-five diagrams. E. B. Treat & Co., 241-243 West Twenty-third street, New York; 199 Clark street, Chicago. 700 pages, price, cloth, $3 net.

This annual has been issued for twenty consecutive years and its publishers claim for it a constantly increasing popularity with the profession. It has grown from a volume of 300 pages to one of more then twice that number. The 1902 number is designed to review the important advances made in practically every branch of medical science during the past year, and considering the broad field covered, this object is accomplished in a much more thorough manner than one would think possible in a work of 700 pages. Important operations and new methods of treatment are considered in detail and, upon all debatable subjects, conflicting views held by authorities are given the prominence they deserve. The parts of the work dealing with materia medica and therapeutics, general medicine and surgery, X-rays, and sanitary science are all that could be desired. Every physician should be familiar with the facts set down in these sections. R.

